# Notes and News

**Published:** 1897-08-14

**Authors:** 


### NOTES AND NEWS.

(Collected and Communicated)

Mr. A. Cameron Skinner has been appointed secretary of the Victoria Hospital for Children in the place of the late Captain Blount.

Liverpool Hospital Sunday Fund amounted to £6,028, and the Hospital Saturday Fund to £5,726 for the present year.

A scheme to erect a hospital for inebriates is entertained at Moscow. The Society of Neurologists and Psychologists of the city have appointed a committee to report on the present state of inebriety there.

At the annual meeting of the Liverpool Convalescent Home the endowment of two beds was announced by Mr. Henry Tate, and a further sum for the re-drainage of the home by the same gentleman.

Beri-Beri still being prevalent at the Richmond Asylum, Dublin, it has been decided to ask the Lord Lieutenant to place Grangegowan Prison at the disposal of the governors until the asylum can show a clean bill of health.

The memorial-stone of a chapel for the Northern Infirmary, Inverness, has been laid by Lady Tweedmouth. The building is to be erected by her at a cost of £2,000, in memory of the late Lord Tweedmouth. The chapel is so arranged that services in connection with various creeds can be held in it.

The following candidates have successfully passed the recent examination in London for commissions in the Army Medical Staff: H. O. B. Browne-Mason, B. Watts, H. G. Martin, F. S. Penny, S. de C. O'Grady, T. H. Gloster, J. G. Berne, A. H. O. Young, J. E. S. Old, F. F. Carroll, J. D. G. Macpherson, W. P. Gwynn, C. J. O'Gorman, M. M. Lowsley, N. H. Ross, E. A. Bourke, A. C. Lupton, P. H. Collingwood, and G. B. Carter.

In America, our claim that we afford facilities for foreign medical men to practise in this country is contradicted. It is held that he is so hampered by restrictions that it is practically impossible for foreigners not holding English qualifications to practise in England; and that it is actually as prohibitive as will be the case in respect to English and other foreign doctors, if the proposed new regulations in Italy are carried into effect.

Diphtheria has shown some increase in London during the past week.

Lord Stanley has opened the new wing at the Rochdale Infirmary, which has been recently added to it. At the opening ceremony special thanks were rendered to the Holden Trustees, who subscribed £20,000 towards the improvement of the infirmary. Mr. Crowther gave £1,000, and Colonel Royds, Mr. S. Turner, and Alderman Duckworth each endowed a bed.

The Duke and Duchess of Connaught visited Portsmouth on Saturday last, and laid the foundation-stone of the first block of the new hospital. The vacant space on the site of the present hospital is to be utilised for the erection of the new building to contain 80 beds. The cost is estimated at about £19,300. The town was decorated in honour of the Royal visit.

The Chemists' Exhibition, though only initiated three years ago, has already attained such developments that its organisers, the proprietors of the *British and Colonial Druggist*, have found it necessary to take Covent Garden Theatre in order to give adequate space to all their exhibitors. The exhibition will be open from the 16th to the 20th of this month, from one p.m. to ten p.m., and promises to be still more interesting and complete than last year. Music and other attractions will be provided for the entertainment of visitors.

Sir Arthur Sullivan has forwarded to the Prince of Wales's Hospital Fund for London a cheque for £202, representing the sum paid by Messrs. Eyre and Spottiswoode in respect of royalty on the sales of the Bishop of Wakefield's Jubilee Hymn, of which the copyright had been assigned to him by the author. Sir Arthur Sullivan, in making this generous donation, expressed the wish that it should be considered a joint gift from the Bishop of Wakefield and himself, as their share of the proceeds of the Jubilee Hymn. In connection with this gift we regret to announce the death of the Bishop of Wakefield, which occurred since this presentation was made.

The Executive Committee of the American Victoria Jubilee Fund report that the lists for endowing beds in hospitals are now closed, and the total obtained amounts to more than £4,300. The surplus to be given to the Prince of Wales's Fund will remain open for the present, and donations to this will be acknowledged by the hon. treasurer, Walter H. Burns, Esq., 22, Old Broad Street. Mrs. Ralph Vivian having written to Sir Fleetwood Edwards asking him to bring to the notice of the Queen the fund which had been collected in her honour, and to participate in commemorating the sixtieth anniversary of Her Majesty's reign, has received the following reply: From Sir Fleetwood Edwards to Mrs. Vivian.—"I have laid your letter of the 25th inst. before the Queen, who desires me to say how touched she is, and also how grateful to yourself and to all those who have contributed to and collected the American Victoria Jubilee Fund. Her Majesty is deeply sensible of the kindness thus evinced, and greatly pleased that the handsome sum thus realised is to be devoted to so good and useful a purpose as the endowment of the beds in the hospitals you name; while the surplus is to be devoted to the Prince of Wales's Fund. The Queen trusts you will be kind enough to express her sincere thanks to all those concerned for their very kind action."

